# Forestry Digital Twin With Machine Learning in Landsat 7 Data

**DOI:** 10.3389/fpls.2022.916900

**Published:** 2022-06-13

**Authors:** Xuetao Jiang, Meiyu Jiang, YuChun Gou, Qian Li, Qingguo Zhou

**Affiliations:** ^1^School of Information Science and Engineering, Lanzhou University, Lanzhou, China; ^2^Shaanxi Province Climate Center, Xi'an, China

**Keywords:** digital twin, remote sensing, machine learning, spatial temporal prediction, Landsat 7

## Abstract

Forest succession analysis can predict forest change trends in the study area, which provides an important basis for other studies. Remote sensing is a recognized and effective tool in forestry succession analysis. Many forest modeling studies use statistic values, but only a few uses remote sensing images. In this study, we propose a machine learning-based digital twin approach for forestry. A data processing algorithm was designed to process Landsat 7 remote sensing data as model's input. An LSTM-based model was constructed to fit historical image data of the study area. The experimental results show that this study's digital twin method can effectively forecast the study area's future image.

## 1. Introduction

Forests are vital to ecosystems. Researchers could discover correlations in the area over time by analyzing changes in forest lands. Time series data analysis of forests makes ecological decision-making more efficient and reliable (Powell et al., 2013). The advancement of computer technology has provided researchers with many valuable tools that allow them to model and analyze data more accurately. Digital modeling has become an attractive and practical topic in several fields. Digital twin (DT) represents the digital modeling of a real-world object (Negri et al., 2017). Theoretically, the study of the DT model is equivalent to the research on the corresponding actual object. If DT technology is applied to forestry, the study area can be approximated using a digital model.

We adopt some feasible approaches from a review of agricultural DT (Cor et al., 2021) because there is no forestry DT review article in our survey. Agricultural DT uses images and point-cloud data from agricultural machines and drones, necessitating the use of a powerful computer for data processing (Polák et al., 2021). Using remote sensing images to analyze forest land could be done on a personal computer. The agricultural DT requires the edge computing devices on farms for real-time monitoring (Nasirahmadi and Hensel, 2022); however, forest lands do not have such conditions, making it difficult to obtain additional forest data. Therefore, remote sensing images provide crucial data in forestry DT. Remote sensing images are used as a widely recognized tool for land surface characterization in forestry analysis, including forest disturbance prediction (Buma and Livneh, 2017), tree species analysis (Fricker et al., 2015), and forest canopy analysis (Joshi et al., 2006). Xuebin et al. (2021) constructed a forest map of the southern Great Plains using data from multiple satellites. Healey et al. (2018) used a random forest algorithm to measure forest variation. Ewa et al. (2020) used models such as XGB (Chen and Guestrin, 2016) to predict forest species. However, no research has been conducted on spatial-temporal prediction of forest remote sensing images, i.e., predicting future forest images using historical image data. Therefore, we propose a long short-term memory (LSTM, Hochreiter and Schmidhuber, 1997) based method to achieve forest image and forest prediction twins.

**Figure 1:**
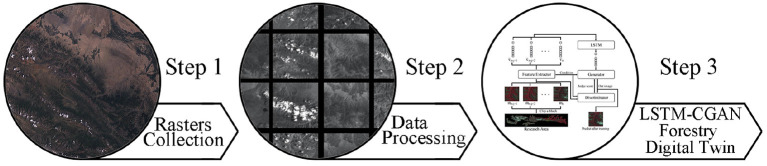
Project work flow.

We obtain United States geological survey (USGS) Landsat 7 data from 2001 to 2021 at yearly intervals for this study (US, 1974). QGIS (QGIS Development Team, 2009) processes multi-view remote sensing images over a year to obtain a complete image of the study area. We use the cropping algorithm to convert high-resolution images into small blocks to minimize the depth of the machine learning model. Finally, the LSTM-based machine learning model is constructed to predict future image data from historical remote sensing image data. The project's process diagram is given Figure 1.

This study is organized as follows: Section 1 introduces the advantages of the forest DT and present the corresponding implementation method. Section 2 formulates the forestry image DT problem and presents the details of the LSTM-based model. Section 3 analyzes the results to verify our method for forest DT.

## 2. Method

### 2.1. Data Processing

**Figure 2:**
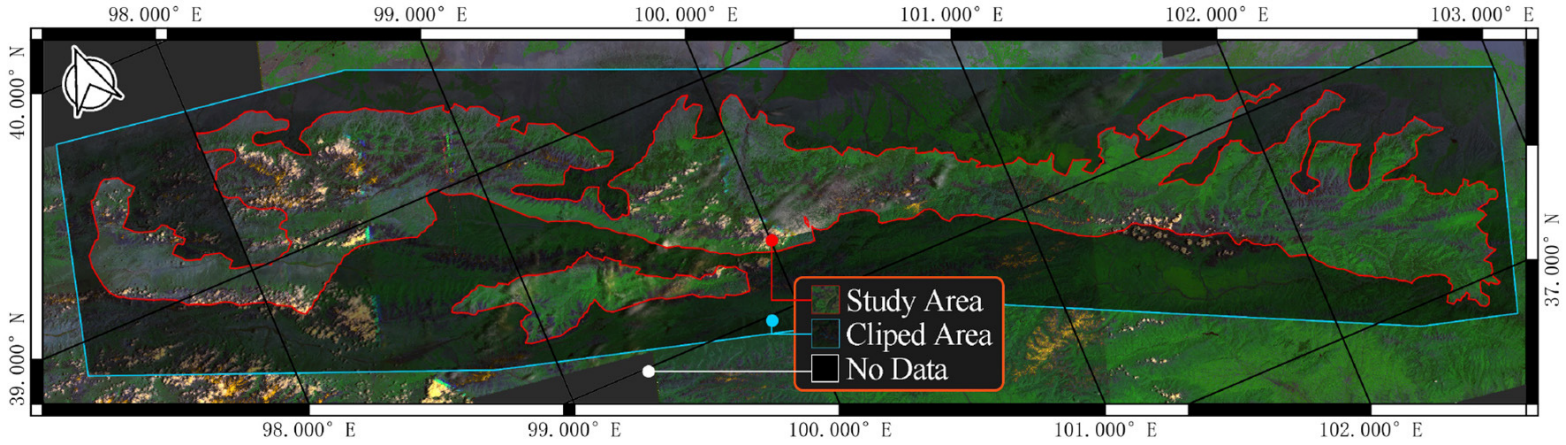
The study area.

The study area is a forest-steppe nature reserve in Gansu Province, China (Figure 2) . We begin by obtaining the area's boundary from the government's website. In USGS Earth Explorer, we choose Landsat 7 ETM + C2 L1 data set for this work. Five views of data were selected according to the boundaries, and their WRS coordinates are 132034, 133033, 134033, and 135033. Then, we collect 20 years of remote sensing data from 2001 to 2021.

Five data views are included every year due to the reserve's size. For each year, the images are filled (gap-filling), merged, and clipped using QGIS to obtain the complete study area from GDAL library (GDAL/OGR Contributors, 2020) and GRASS7 library (GRASS Development Team, 2017). Finally, 21 year-by-year large remote sensing images were obtained. The code for data processing can be found on https://github.com/JChrysanthemum/ForestyDT/tree/datatprocess, and the processed data can be found on https://www.kaggle.com/datasets/jchrysanthemum/qmnp-landsat7?select=Dataset.

**Figure 3:**
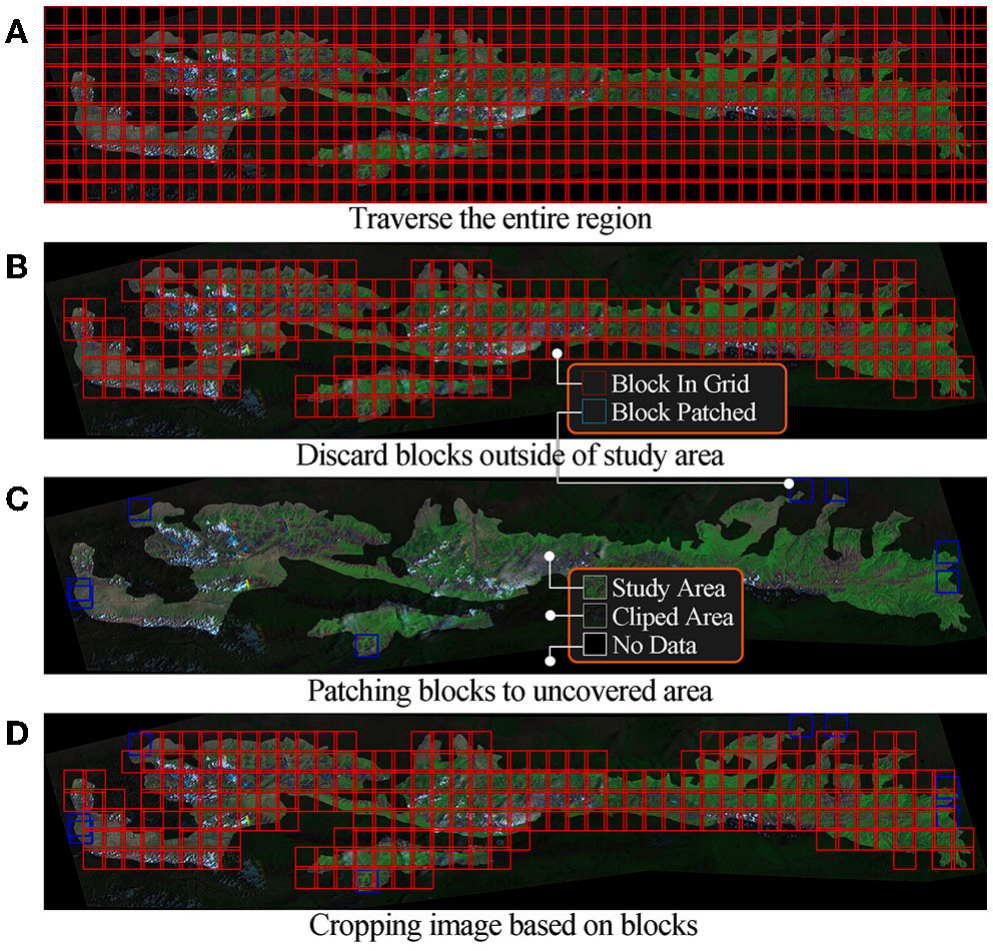
Cropping remote sensing images.

However, modeling using the above images may suffer from insufficient data, oversized models, and low accuracy. We use a cropping algorithm to convert large images into smaller ones. As show in Figure 2, the clipped area is an extension of the study area to ensure that the machine learning model can use all pixels in the study area. Describing these algorithms may take up a lot of space since the cropping algorithm in this study uses tricks such as sliding windows, variable window size, and joint domain detection. The key steps of the algorithm are represented in Figure 3.

We first traverse the entire region based on the cut size and move steps, as shown in Figure 3A. Adjacent data blocks have overlaps designed to smoothen the final prediction. We discard data blocks that do not contain any pixels from the study region after traversal, as shown in Figure 3B. We use a gradient ascent strategy to discover data blocks in uncovered areas of study regions in as few steps as possible, and the results are shown in Figure 3C. The coordinates used for cropping are the algorithm's output, as shown in Figure 3D. Finally, we crop the images of each band to generate a dataset, which is used for subsequent model training. The dataset contains 42,840 images from 8 bands. Each one is a grayscale image with a size of 128 × 128 pixels.

### 2.2. Forest Image DT Formulation

We provide a formulaic description of the forest image DT in this section, which serves as the theoretical basis for the model design. Forest image DT uses historical remote sensing data for modeling, which predicts later frames using former frames. Reducing the data variability sequentially can improve the robustness of models. We calculate the mean and variance of the images over 20 years for each block and apply max-min regularization to them. The regularized input image can be represented as a square matrix *m* ∈ **R**^*n*^. Suppose the total frames of observable data is *n*, where *j* consecutive observations are denoted as ${\tilde{m}}_{n-j+1}\cdots {\tilde{m}}_{n}$. The first n + 1 data, *m*_*n*+1_ is unobservable and the corresponding maximum estimate is denoted as ${\hat{m}}_{n+1}$. Then, the prediction of the next data from j consecutive observations can be expressed as follows:


(1) $$\begin{array}{l} {\hat{m}}_{n+1}=\underset{m_{n+1}}{\mathrm{argmax}}P\left(m_{n+1}|{\tilde{m}}_{n-j+1}\cdots \;{\tilde{m}}_{n}\right) \end{array}$$

### 2.3. Forest Image DT Model

Our model consists of three components: a feature extraction network, a LSTM, and a generative network. The feature extraction network uses a convolutional structure to downscale the remote sensing image data. The LSTM uses several downscaled neighboring frames as input, then outputs the feature vectors of the predicted frames. The generative network generates the predicted frames using the features obtained from the LSTM network.

#### 2.3.1. LSTM Model and Feature Extraction Model

The order of observable historical images $\tilde{m}$ is 16,384 for each block. It has low dimensionality as a picture but high dimensionality as a time series. Therefore, we used a two-layer convolutional network, *f*, as feature extraction network to reduce the dimensionality of each image data. In our experiments, the training of the other models became unstable without a feature extraction network. The training loss converges very slowly if the images are input directly to the LSTM network for temporal feature extraction.


(2) $$\begin{array}{l} {ṽ}_{i}=f\left({\tilde{m}}_{i}\right),i\in n-j+1\cdots \;n \end{array}$$

The dimensionality of feature vector *v* is significantly smaller than the image $\tilde{m}$ due to the feature extraction network, *f*. We use the LSTM model to extract the temporal features from the reduced vectors in the next step. LSTM is a special recurrent neural network that is good at learning the dependencies from long sequences. The cell state is the LSTM's key structure, as it controls whether the newly added state is forgotten or retained. The LSTM outputs the final state by accepting several consecutive historical data.


(3) $$\begin{array}{l} z=lstm\left({ṽ}_{n-j+1},\cdots \;,{ṽ}_{n}\right) \end{array}$$

The final state, *z*, obtained from LSTM through vectors *ṽ*_*n*−*j*+1_ ⋯ *ṽ*_*n*_ is the temporal feature vector for a generative adversarial network (GAN, Goodfellow et al., 2014) model.

#### 2.3.2. Generator Model

GAN could generate new data similar beyond the original data set, consisting of a generator and discriminator. However, GAN product new images, not reproduce images in data set. The new images generated by GAN have the features of several images in the dataset but may not be similar to a particular image. We want the predicted image in our DT model to be as similar to the actual image as possible. Therefore, we only used the generator in GAN and optimized it with the other models. We use the design of conditional GAN (CGAN) in the model since the predicted images are divided into many blocks (Mirza and Osindero, 2014). We use the data coordinates as the conditions, e.g., 0111 denotes the seventh block in the grid. Our method can model many blocks at the same time by incorporating the additional condition.

#### 2.3.3. Model Design

**Figure 4:**
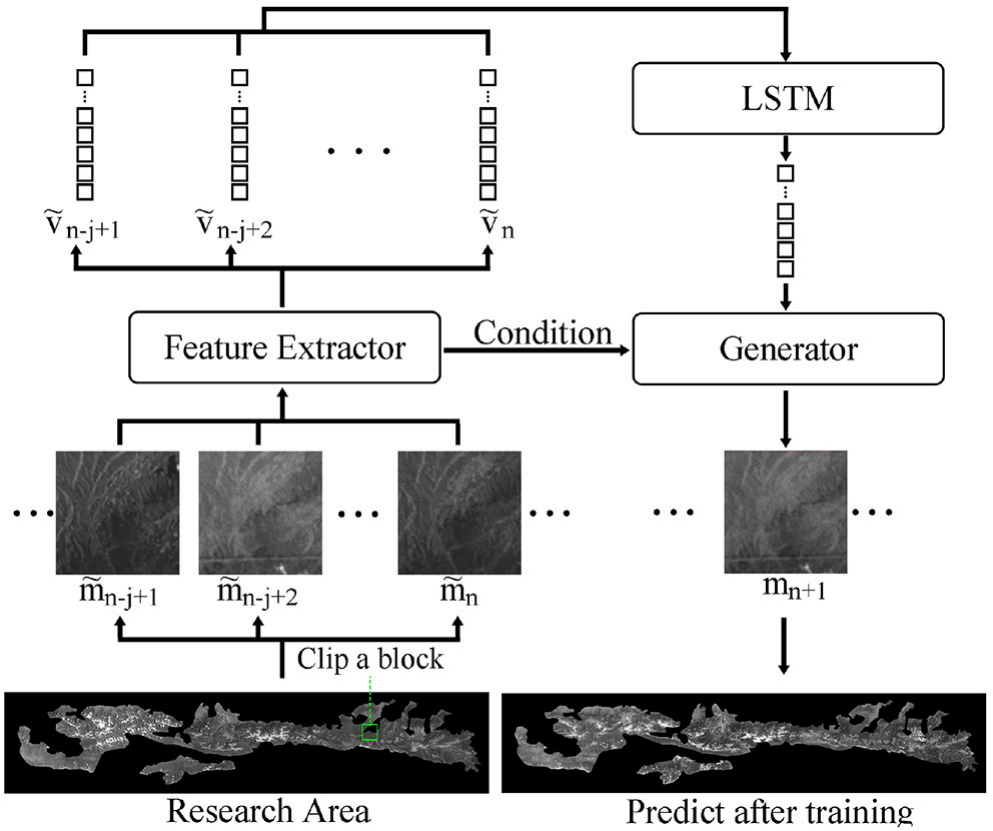
Data flow of the model.

The model in this study consists of the aforementioned three parts as shown in Figure 4.

*m*_*n*+1_ can be replaced by *G*(*z, c*) in Equation 1 since we use a generator to rebuild future image. Next, the feature extraction network extracts downscaled ${\tilde{m}}_{n-j+1}\cdots {\tilde{m}}_{n}$ into *ṽ*_*n*−*j*+1_ ⋯ *ṽ*_*n*_. Feature vectors, *ṽ*_*n*−*j*+1_ ⋯ *ṽ*_*n*_, are associated with the generating condition, *z*, using the LSTM model. The final result is an approximation of the actual image, achieving the goal of the DT model. Finally, equation 1 can be expressed as follows:


(4) $$\begin{array}{l} {\hat{m}}_{n+1}=\underset{z}{\mathrm{argmax}}p\left(G\left(z\right)|{\tilde{m}}_{n-j+1}\cdots \;{\tilde{m}}_{n}\right)\\ \;\approx \underset{z}{\mathrm{argmax}}p\left(G\left(z\right)|{ṽ}_{n-j+1}\cdots \;{ṽ}_{n}\right)\\ \;\approx G\left(lstm\left({ṽ}_{n-j+1},\cdots \;,{ṽ}_{n}\right)\right) \end{array}$$

### 2.4. Model Training

Many researchers also use the generator as part of the model, which has two different training methods. The first method trains the GAN alone, then combines the pretrained generator with the other models for training. The second method trains the generator with the other models simultaneously. We tried the first method several times, but overall model's training became unstable and collapse frequently. As for the second method, it behaves more robustly. Since embedded GAN training is not the focus of this work, we only use the second approach. Then, we added a parametric Gaussian distribution layer to the end of LSTM, as the GAN model input requires a Gaussian distribution.

Using the feature extraction network *f*, LSTM network *lstm*, generator network *G*, training epochs *itr*, and data set *ds*, as inputs, Algorithm 1 outputs the trained model in the end. Lines 1–10 indicate the model's *itr* training times. Line 3 indicates that the input matrices are compressed into feature vectors by the feature extraction network. Line 4 indicates that the feature vector is transformed into a temporal feature sequence through the LSTM. Lines 6–9 indicate that the three networks are trained sequentially by back-propagating the loss.

**Algorithm 1:**
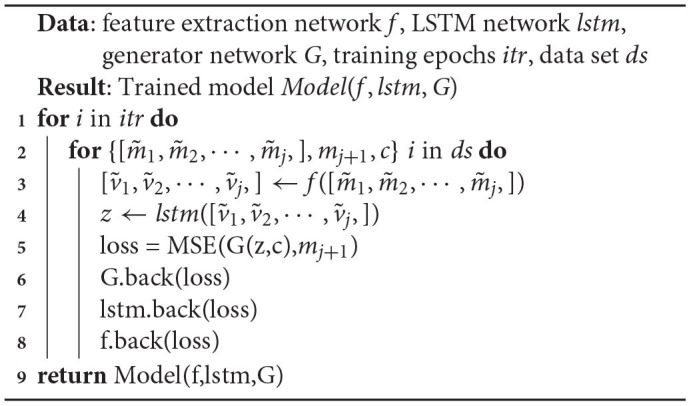
Model training

## 3. Result and Analysis

The analysis of remote sensing images requires several different band combinations. Here, we use a B432 combination as an example. In this study, data from 2001 to 2020 were used for training, and data from 2021 were used for prediction. In addition, we test four classical models: LSTM Wasserstein-GAN (WGAN) model (Xu et al., 2019), convolutional LSTM model (Liu et al., 2017), auto-encoding (AE) CGAN model and AE WGAN model (Makhzani et al., 2016). The code of all models can be found on https://github.com/JChrysanthemum/ForestyDT/tree/main

The models take data from 2018-2020 as input, resulting in 255 blocks for 2021. Second, the blocks are merged to obtain three band images of study area. Then, the band images are stacked to get false-color images, which are used for feature detection. For simplicity, we get the vegetation segmented image from false-color image by color. In the following, we will evaluate the predicted blocks and the segmented images.

First, we evaluate blocks using normalized root mean square error (NRMSE) to measure their similarities. Where *y*_*i*_ denotes the true value, ȳ denotes the mean of the true value, $\hat{y_{i}}$ denotes the predicted value, and σ denotes the standard deviation.


(5) $$\begin{array}{l} NRMSE=\sqrt{\frac{\sum_{i=1}^{n}{\left(y_{i}-ŷ\right)}^{2}}{n\sigma^{2}}},\;RMSE=\sqrt{\frac{\sum_{i=1}^{n}{\left(y_{i}-ŷ\right)}^{2}}{n}} \end{array}$$

Next, we use the correct rate (CR) to measure the overlap between predicted and true segmented images. *num*_*hit*_ indicates the number of correctly predicted pixels, while *num*_*miss*_ is the opposite.


(6) $$\begin{array}{l} CR=\frac{num_{hit}}{num_{hit}+num_{miss}} \end{array}$$

**Figure 5:**
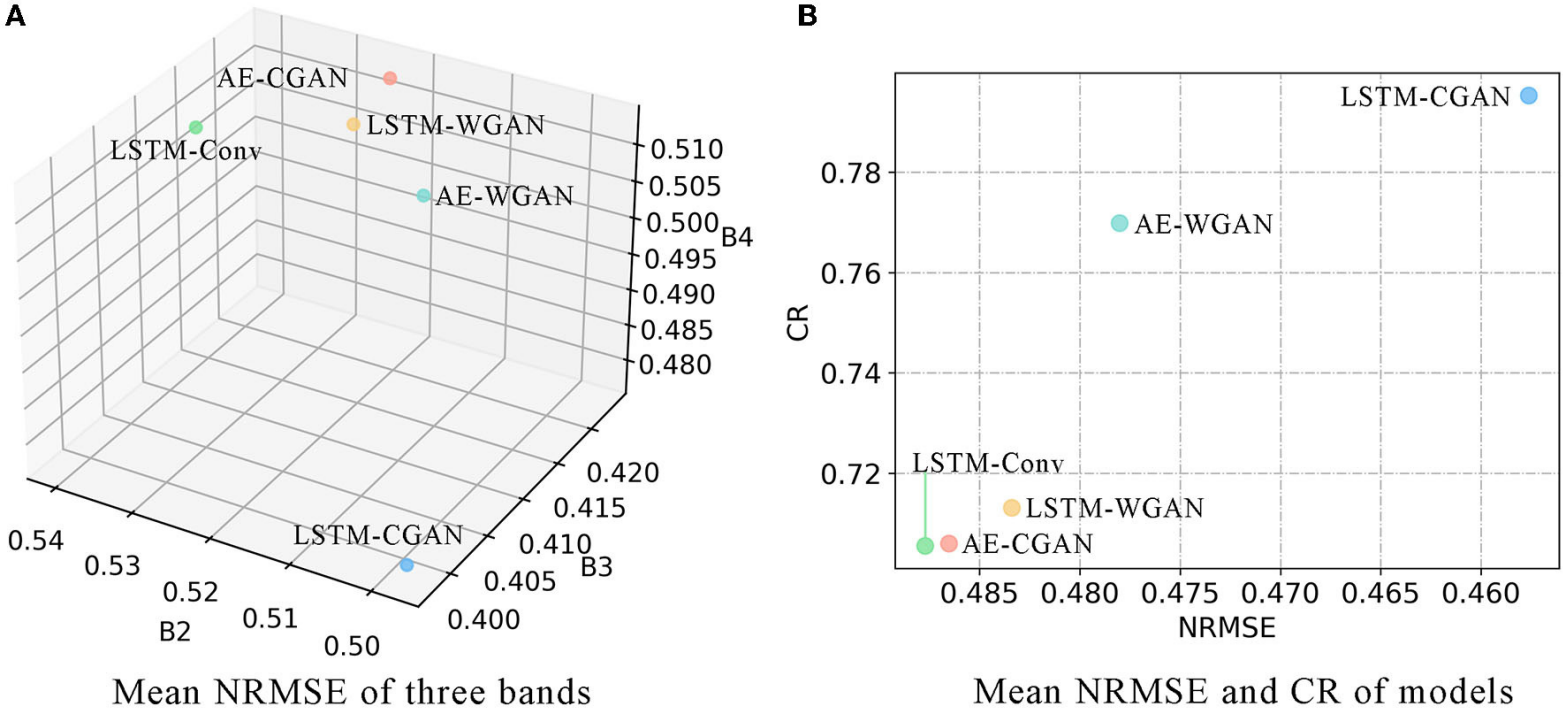
**(A,B)** NRMSE and CR of models.

For each band of 255 blocks, we calculate the NRMSE between predicted and true values. Then derive the mean and standard deviation of NRMSE scores. For the segmented images, we calculated their CR. The results are shown in Table 1 and Figure 5.

**Table 1:** Model scores comparison.

**Model**	**NRMSE (Mean, Std Dev)**			**CR**
	**B2**	**B3**	**B4**	**B432**
**LSTM-CGAN**	(**0.50**, **0.23**)	(**0.40**, **0.17**)	(**0.48**, 0.17)	**0.80**
LSTM-Conv	(0.54, 0.30)	(0.41, 0.19)	(0.51, 0.17)	0.71
LSTM-WGAN	(0.52, 0.27)	(0.42, 0.19)	(0.51, **0.16**)	0.71
AE-CGAN	(0.52, 0.25)	(0.42, 0.18)	(0.51, **0.16**)	0.71
AE-WGAN	(0.51, 0.25)	(0.42, 0.18)	(0.51, **0.16**)	0.77

Figure 5A plots the mean NRMSE for three bands, and Figure 5B plots the mean NRMSE and CR. Obviously, most scores of LSTM-CGAN are optimal. Next, we further analyze the predicted images of LSTM-CGAN.

**Figure 6:**
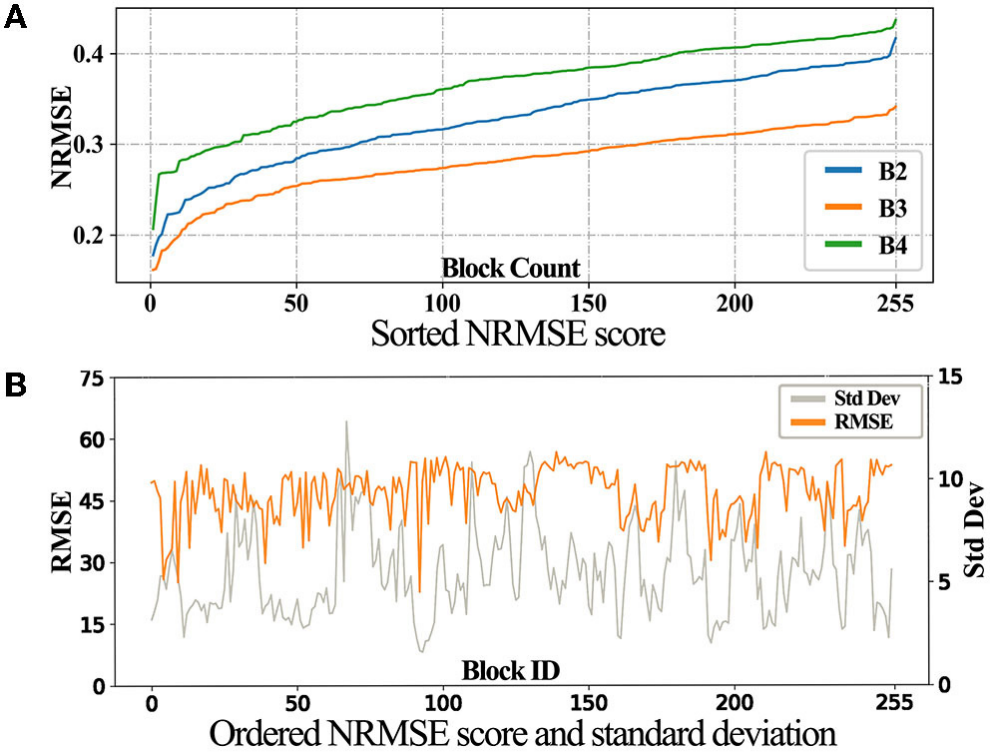
**(A,B)** LSTM-CGAN scores.

We sort the RMSE score for these images, as shown in Figure 6A. Taking B3 as an example, we derived the standard deviation of each block over 20 years. Note that we use RMSE instead of NRMSE, because NRMSE are affected by the standard deviation. Then, the standard deviations and the corresponding RMSE scores are plotted as shown in Figure 6B.

In Figure 6A, the three curves displayed the same trend, with B3 being the best. In Figure 6B, the trend of NRMSE is opposite to the standard deviation, showing that the temporal stability of the images affects the final prediction.

**Figure 7:**
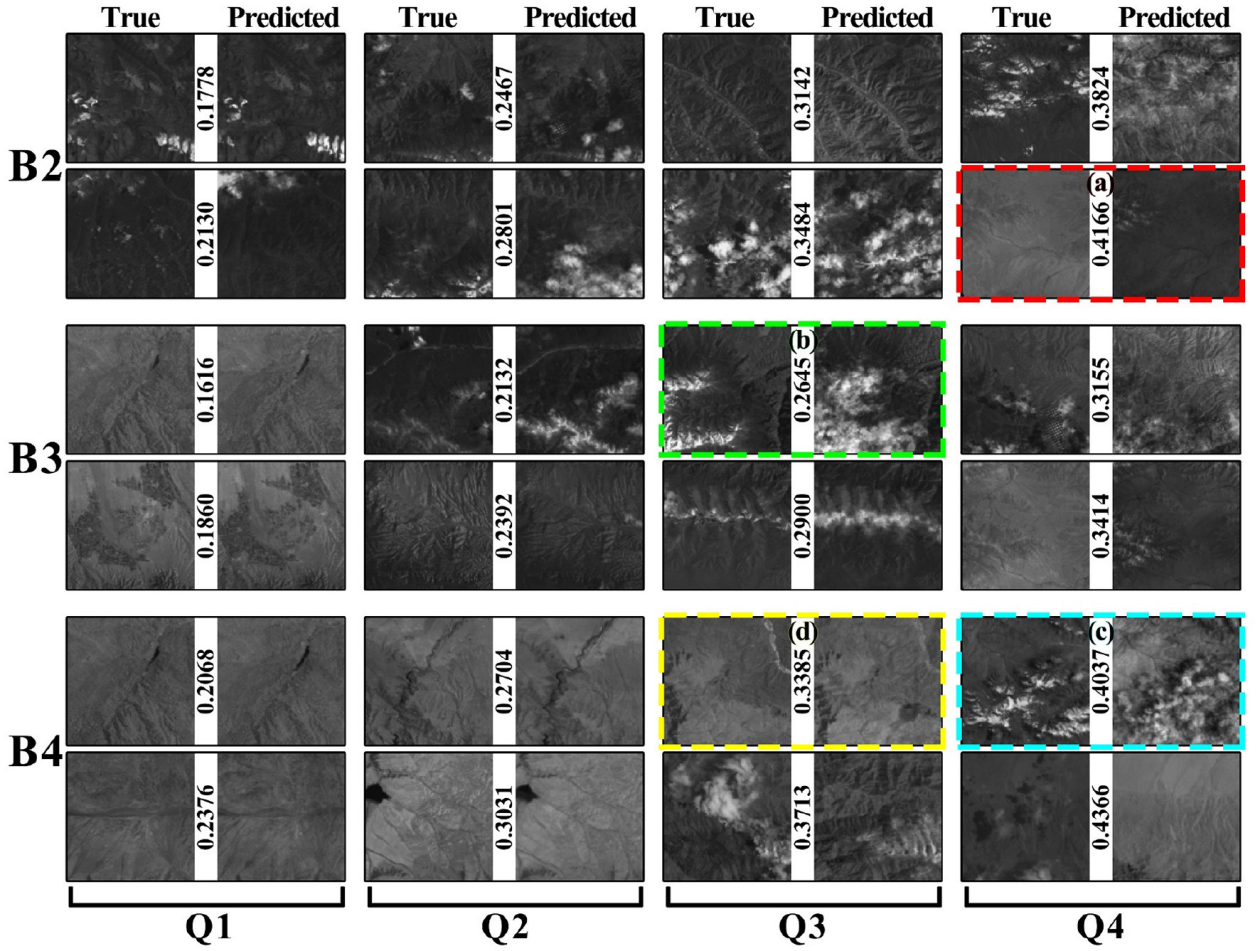
NRMSE scores and the prediction.

We group the blocks based on the NRMSE scores in Figure 7 to reveal inaccurate prediction cases.

Figure 7 shows eight pairs of images for each band, which are equally divided into four intervals. For each pair of images, the left one is the true value, and the right one is the predicted image. A low score indicates a high similarity between the two images. The predicted and true values are similar in columns Q1 and Q2. The predictions in columns Q3 and Q4 are biased but still have some common regions with true value. We selected four typically dissimilar conditions, indicated by colored dashed boxes in Figure 7. Figure 7A shows that the prediction and true values are not in one color interval. This block had different color distributions from 2018 to 2020. This may result in bias in the model's prediction interval. Figure 7B shows that the prediction has an additional cloud. It is almost impossible to guarantee that every piece of data in remote sensing data with a long-time span over large areas is cloud-free. The cloud coverage model in a region cannot be built if meteorological data for the region are not available. Figure 7C is the simultaneous occurrence of cases (Figures 7A,B). Figure 7D shows that the changes in the region are not reflected in the prediction. For this region, the changes that occurred in 2021 did not occur in the previous years. Therefore, the inconsistency between the predicted and true values may be influenced by the inconsistency in the data. It is difficult to optimize the model to get a better prediction without meteorological data such as local light radiation data.

**Figure 8:**
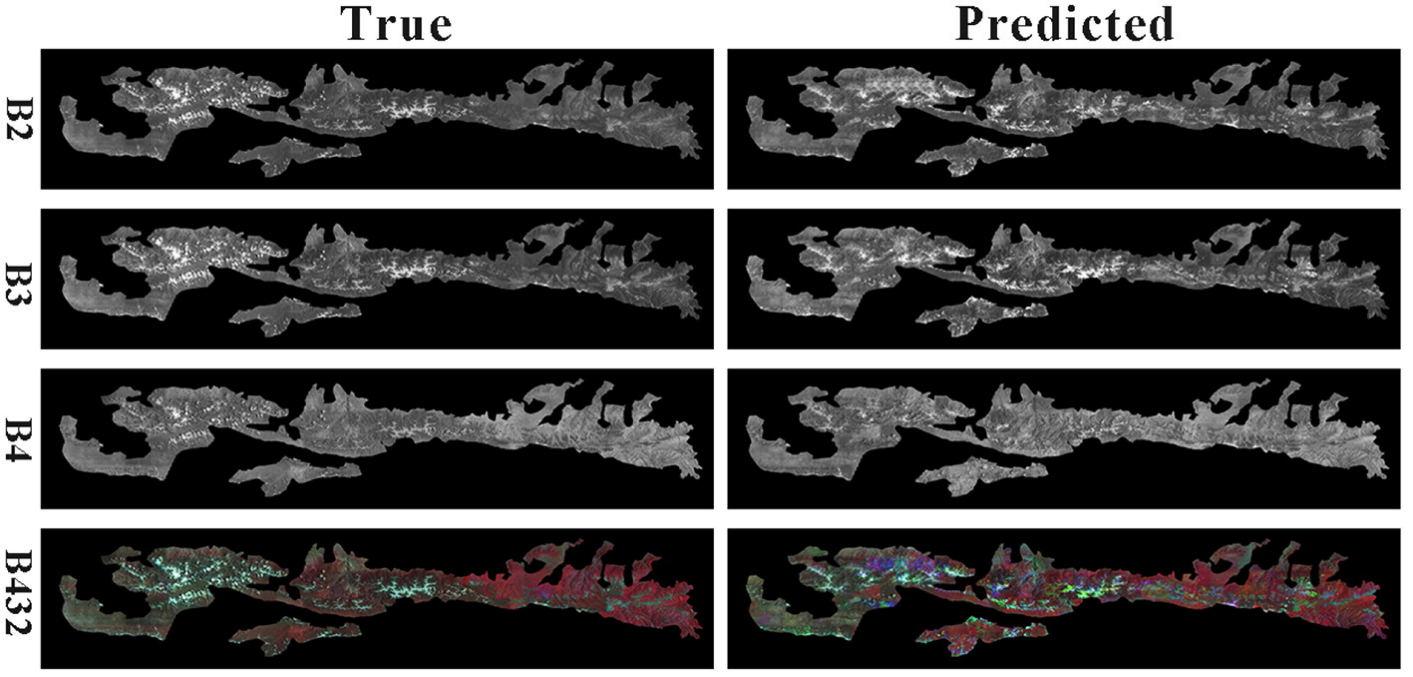
Model prediction for B2, B3, B4, and B432.

The final result comparison is shown in Figure 8.

From the figure, the generated image is similar to the predicted image. It proves that the method in this paper achieves a digital twin of the forest, which provides a basis for other studies by predicting future remote sensing images.

## 4. Conclusion

In this paper, a digital twin method for forestry image prediction is designed to predict future remote sensing data by using historical Landsat 7 remote sensing data. A forestry image cropping algorithm was designed to reduce the large-scale remote sensing images into smaller blocks according to the study area to model large-scale forestry images. An LSTM-based model was designed to predict future remotely sensed images using remote sensing image time series for training. The prediction results show that the method can predict the development of forestry images to a certain extent and works effectively as a forestry prediction twin. Our method achieves a digital twin of large areas of forest that can predict future remote sensing images. This allows forestry canopy, species and distribution succession to be obtained from images, making the analysis more direct and concrete.

## Data Availability Statement

The original contributions presented in the study are included in the article/supplementary material, further inquiries can be directed to the corresponding author/s.

## Ethics Statement

Ethical review and approval was not required for the animal study because this is a new work that unfolds a new study of forestry remote sensing images through artificial neural networks. No other expert with both AI and forestry remote sensing knowledge has been found for review.

## Author Contributions

All authors listed have made a substantial, direct, and intellectual contribution to the work and approved it for publication.

## Funding

This work was supported by Shaanxi Province Natural Science Basic Research Project (2019JQ-990).

## Conflict of Interest

This study received funding from QL. The funder had the following involvement with the study: data interpretation and collection. The remaining authors declare that the research was conducted in the absence of any commercial or financial relationships that could be construed as a potential conflict of interest. The handling editor JS declared a past collaboration with the author QZ at the time of the review.

## Publisher's Note

All claims expressed in this article are solely those of the authors and do not necessarily represent those of their affiliated organizations, or those of the publisher, the editors and the reviewers. Any product that may be evaluated in this article, or claim that may be made by its manufacturer, is not guaranteed or endorsed by the publisher.
